# Editorial: Current perspectives in urology education

**DOI:** 10.3389/fruro.2023.1272810

**Published:** 2023-09-11

**Authors:** Lauren E. Corona, Kate H. Kraft, Christopher Jaeger, Tasha Posid, Benjamin N. Breyer

**Affiliations:** ^1^ Ann & Robert H. Lurie Children’s Hospital of Chicago, Chicago, IL, United States; ^2^ Department of Urology, University of Michigan, Ann Arbor, MI, United States; ^3^ Department of Urology, Boston Children's Hospital, Harvard Medical School, Boston, MA, United States; ^4^ Department of Urology, The Ohio State University, Columbus, OH, United States; ^5^ Department of Urology, University of California, San Francisco, San Francisco, CA, United States

**Keywords:** education, training & development, residency and fellowship, technology, didactic, evaluation, surgical skill assessment

Advances in technology, new approaches to surgical training, updated accreditation requirements for graduate medical education, and shifting perspectives in trainer/trainee dynamics have contributed to a changing landscape in urology education over the past decade. These changes are highlighted nicely in the contributions to this “*Current Perspectives in Urology Education*” Research Topic.

Sheetz et al. published their work titled “*Implementation and assessment of a novel non-clinical skills curriculum for urology residents.*” In this pilot study, the authors implemented and evaluated a non-clinical skills curriculum for urology residents. The study aimed to address the need for formal training in the often-overlooked hidden curriculum of leadership and professional development. Their curriculum consisted of a series of monthly workshops conducted by local experts that focused on improving non-clinical subject knowledge covering a range of topics. Eighteen urology residents participated over two academic years. A needs assessment revealed that such a curriculum was desired by the residents. Upon completion of the curriculum, results boasted a 17% and 21% increase in subject knowledge for senior and junior residents, respectively. The authors concluded that implementation of a non-clinical skills curriculum for urology residents was desired, feasible, and well-received by residents. Most importantly, the curriculum led to significant improvements in non-clinical subject knowledge.

In “*Evaluation of Urology Trainee Preferences in Didactic Education: A Choice-based Conjoint Analysis*,” authors Li et al. sought to identify the educational preferences of urology trainees. Urology trainees from across the country completed an online survey assessing combinations of four attributes: mode of communication, presentation style, presenter credentials, and curriculum design. Results showed a distinct preference among trainees for online/virtual presentations by national experts in a case-based format in the form of a nationally standardized curriculum. Preferences varied based on level of training. These findings suggest the importance of incorporating online didactics and collaborative resources into urology residency curricula to meet the needs of trainees.

A review of the literature by Yelton at el. titled “*Evaluating the effect of the COVID-19 pandemic on the use and impact of social media in the urology residency match*” was conducted to explore the impact of social media on the urology residency application and match process in the U.S. The review included 9 sources comprising peer-reviewed articles, conference abstracts, and a research letter. Results consistently indicated an increased use of professional social media amongst both urology applicants and residency programs, particularly on Twitter. Applicants found social media useful for gathering information about programs and connecting with residents, faculty, and program directors. This finding stood out vis-à-vis program directors who rarely reviewed applicants’ social media profiles during the match process and mostly reported that social media had no role in their assessment of applicants. The use of social media is expected to continue even in the post-pandemic world, and ongoing analysis of its benefits and drawbacks are crucial as virtual interactions become increasingly relevant in the urologic community.

Finally, Khoei et al. published their work titled “*Design and Evaluation of a Virtual Urology Sub-Internship During the COVID-19 Pandemic.*” The authors in this study developed and evaluated a 2-week virtual elective course with a curriculum consisting of live surgical case streaming, one-on-one didactics, and in-person clinic virtual participation. Eleven fourth-year medical students participated. Results from a post-participation survey (with a 91% response rate) showed a high level of satisfaction with the educational experience. Live surgery and one-on-one didactics stood out as strengths. The curriculum was economical and found to effectively engage medical students and provided a “prototype” for a virtual rotation that could be adopted by other programs.

These four articles underscore the growing importance of the integration of innovation and technology, simulation, and augmenting education through virtual learning to enhance surgical skills, didactic education, and patient care. Additionally, the articles advocate for competency-based education, emphasizing the assessment of specific milestones and the need for standardized evaluation methods. Finally, the articles highlight the significance of incorporating global perspectives, cultural sensitivities, and diverse surgical techniques into training programs to broaden the scope of urology education and enhance trainees’ understanding of the field’s global challenges and practices. These articles contribute to the urology literature by showcasing the evolving landscape of urology education and training.

## Author contributions

LC: Writing – original draft, Writing – review & editing. KK: Writing – review & editing. CJ: Writing – review & editing. TP: Writing – review & editing. BB: Writing – review & editing.

